# Effect of Different Surface Treatments of CAD/CAM Zirconia Core on the Final Color of Porcelain-Veneered Restorations

**DOI:** 10.34172/joddd.44821

**Published:** 2026-06-30

**Authors:** Ali Barzegar, Mehrnaz Motiei, Tahereh Ghaffari, Peyman Irankhah

**Affiliations:** ^1^Department of Prosthodontics, Faculty of Dentistry, Tabriz University of Medical Sciences, Tabriz, Iran; ^2^General Dental Practitioner, Private Practice, Tabriz, Iran

**Keywords:** Porcelain color, Surface treatment, Veneered restorations, Zirconia

## Abstract

**Introduction:**

Achieving accurate color matching is essential for the aesthetic success of zirconia-based restorations, especially in the anterior region. Surface treatments are commonly used to enhance the bond between zirconia and porcelain, but their impact on the final color perception remains unclear. This study evaluated the effects of three surface treatments (control, acid etching, and sandblasting) on the color characteristics of porcelain-veneered zirconia.

**Methods:**

Sixty zirconia samples were divided into three groups: no surface treatment (control), acid etching, and sandblasting. Color measurements were taken using the CIELab* color system, with a specific focus on the color difference (ΔE) and the individual components of lightness (L), the red-green axis (a), and the yellow-blue axis (b).

**Results:**

The results showed that sandblasting resulted in the highest ΔE value (1.84±0.27), significantly greater than those of the control (ΔE=0.77±0.43) and acid etching (ΔE=0.67±0.22) groups (*P*<0.05). Additionally, the sandblasted group exhibited a significant decrease in lightness (L=76.12), while the control group had a higher L value (L=77.85). No significant difference was observed between the acid-etched and control groups for any color component (*P*>0.05).

**Conclusion:**

Sandblasting significantly altered the optical properties of zirconia, leading to noticeable changes in color, particularly in lightness. In contrast, acid etching had minimal impact on color changes. These findings have clinical implications for the aesthetic planning of zirconia-based restorations, particularly in the anterior region, as highly aesthetic areas, where precise color matching is crucial.

## Graphical Abstract

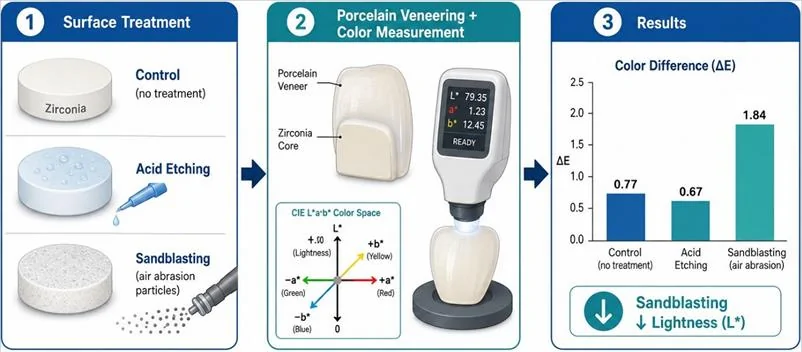

## Introduction

 Compared to ceramics, PFMs have a metal substructure that reduces light transmission through the ceramic, especially when there is insufficient space for veneer materials. Other disadvantages of metal‒ceramic restorations include opaque appearance, gingival discoloration, visible metal margins, and porcelain discoloration.^1^

 Superior mechanical properties, esthetics, and biocompatibility of the zirconia substructure have made it an excellent candidate for dental applications.^2^ Dental zirconia is composed of zirconia tetragonal polycrystals stabilized with yttria (Y-TZP) or partially yttria-stabilized zirconia (YSZ). As Y-TZP3 zirconia is a white, opaque material with low translucency and cannot replicate the optical properties of enamel,^3,4^ restorations with a zirconia core and a porcelain veneer are used.^5^

 Factors responsible for the failure of prosthetic treatment include a weak bond between porcelain veneer and zirconia, low wettability of the zirconia substructure with porcelain, repeated occlusal forces, residual stresses due to different coefficients of thermal expansion, incorrect framework design, thickness of the veneered porcelain, ceramic defects, ceramic contraction from porcelain firing, type of ceramic and its placement technique, and phase transformation of zirconia crystals at the zirconia‒porcelain interface, leading to porcelain veneer chipping.^6^

 A study by Anusavice et al.^7^ demonstrated the strength of zirconia; however, a weak bond between the core and porcelain was observed, leading to ceramic veneer fracture. Therefore, strengthening the aforementioned bond to transfer functional stresses from esthetic veneers to the underlying frameworks is essential.

 Various surface preparations, including laser, silica coating, liner application, polishing, HF acid etching, and air abrasion on ceramic substructures, have been performed to increase wettability, raise surface energy, and increase surface roughness to increase the bond strength between the zirconia core and ceramic veneer.^8,9^

 Multiple factors can affect restoration color, including zirconia thickness and brand,^10,11^ the type and thickness of the cement used,^12^ and the coloring and surface preparation methods.^13^ Color can be evaluated qualitatively (subjectively) or quantitatively (objectively) through visual matching.^14^

 Given that numerous studies^15-18^ have used HF acid etching and sandblasting of zirconia to increase its bond strength to porcelain, and considering that patients’ esthetic demands are increasing, this study evaluated and compared the effects of surface preparations using HF acid etching and sandblasting of zirconia on the final color of restorations.

## Methods

###  Sample Size

 Sample size was calculated using G*power 3.1.9.7 software with the Wilcoxon-Mann-Whitney test, considering a 5% error rate and 80% test power, with an effect size of 0.466 for 3 groups, resulting in 66 samples: the control group (n = 22), HF acid etching group (n = 22), and sandblasting group (n = 22).

###  Sample Preparation

 Raw, uncolored zirconia samples were fabricated using a YUCERA CAD/CAM device (YRC-5X, Yucera, China) and designed in Exocad software. All the samples were sintered according to the manufacturer’s instructions to achieve complete density using a sintering furnace (F2L 1720, Azar Furnaces, Iran). Subsequently, the samples were gradually cooled under controlled conditions to prevent thermal stress and damage to the specimens. Finally, the dimensions of rectangular blocks after sintering were 5 mm in length and width and 10 mm in height.^19^ The dimensions of the sintered samples were checked using a metal gauge caliper (Fattah Teb, Iran). If the dimensions did not match, they were corrected. Samples with bubbles, nodules, and surface defects were excluded from the study.

 Then, the sample surfaces were polished by the researcher using 600-grit silicon carbide paper (MATADOR, German) in one direction and cleaned using an ultrasonic device (Biosonic Jr, Whaledent, USA) containing acetone (Aylar, Iran) and distilled water (Dionizer device model 200M, Pars Kimiya Mavad Company, Iran) for 15 minutes.^16^ Subsequently, the samples were randomly divided into three groups (Figure 1).

**Figure 1:**
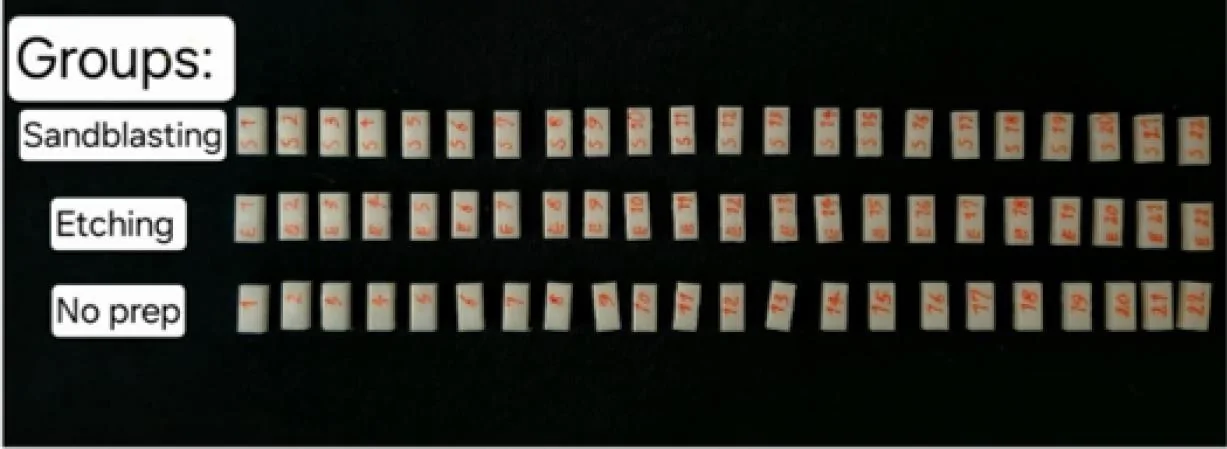
Prepared zirconia samples

####  Group 1

 Control group (without any surface preparation)

####  Group 2

 Surface preparation group using the HF acid etching method. Etching of the samples was performed for 30 minutes according to the manufacturer’s instructions in an etching solution (ZIRCOS-E, BIO DEN CO, Ltd, Korea), which was a mixture of nitric acid, hydrofluoric acid, and several other compounds. The samples were removed from the solution, placed in an ultrasonic bath for 30 minutes, then removed from the bath and placed under running water for 2 minutes. Subsequently, moisture from the samples was removed using a stream of dry air (Figure 2).^16^

**Figure 2:**
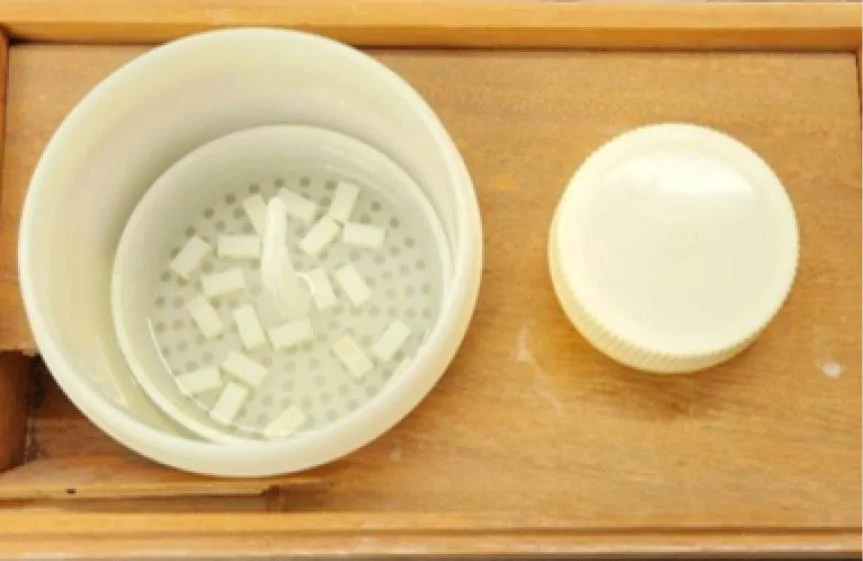
Preparation steps of the etching group

####  Group 3

 Surface preparation group using the sandblasting method. Additional surface preparation was performed by air abrasion with 50-μm aluminum oxide particles (white aluminum oxide, Siladent, Germany) at a pressure of 2.8 bar and from a distance of 10 mm from the sample surface at 10 s/cm^2^ using a sandblasting device (FineBlast, KOUSHAFAN PARS Co., Iran) (Figure 3).

**Figure 3:**
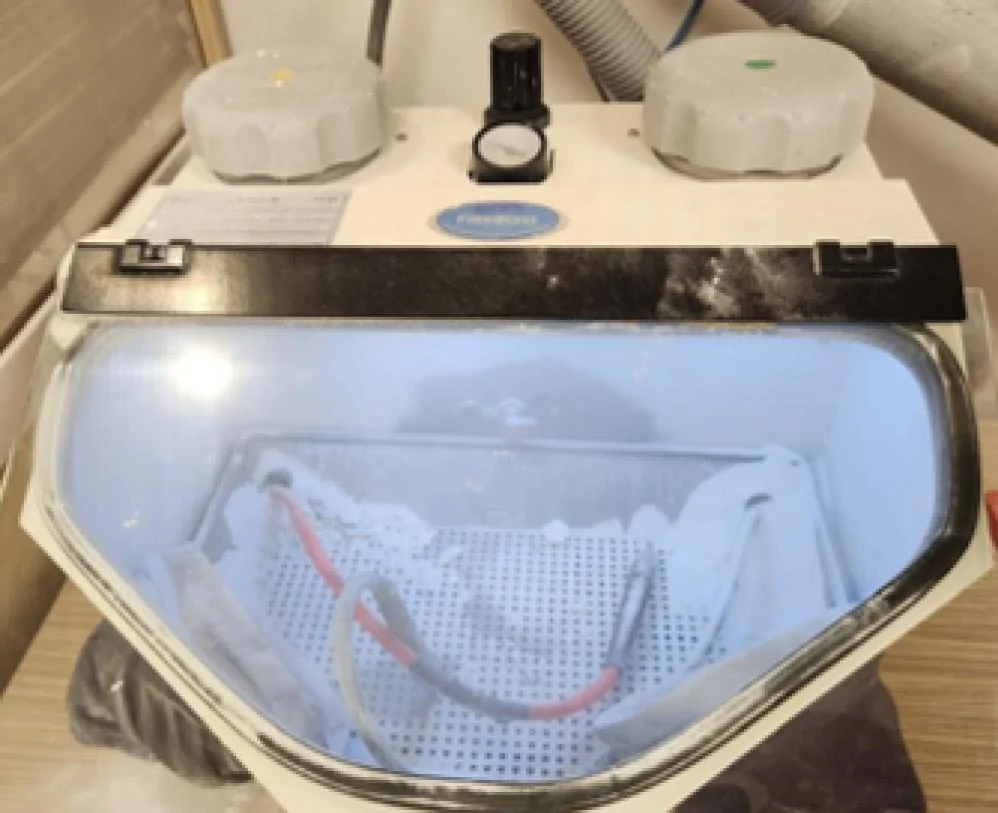
Sandblasting device

 After completing the surface preparation steps, porcelain (Initial GC, Zr-FS, GC, Japan) with A2 color and 2.5-mm thickness was placed on the upper surface of the samples using a metal mold (5 mm in length and width and 12.5 mm in height; with an 0.5-mm estimate of porcelain shrinkage) whose walls were in contact with the zirconia core. Then the mold was removed, and the porcelain on the samples was fired according to the manufacturer’s instructions using an AT300P device (KFP Dental, Iran).^20^

 The final dimensions of the cubic samples after firing were 5 × 5 mm with a height of 12 mm (10 mm zirconia, 2 mm porcelain), as verified with calipers. If the dimensions did not match, they were corrected, and if there were bubbles, nodules, or surface defects, they were excluded from the study.

 The porcelain surface was polished using 400-, 600-, and 1200-grit silicon carbide sandpaper (MATADOR, Germany) in one direction under cold water by one operator. Finally, a porcelain glaze (Glory, Turkey) was applied by firing a single layer onto all samples, following the manufacturer’s instructions.^21^

 After glazing, the final thickness of the samples was measured with calipers, and their color was evaluated with a spectrophotometer (UV-SP62; X-Rite Inc., Grandville, MI, USA). Before measuring the samples’ color, the spectrophotometer was calibrated to the manufacturer’s recommended baseline color value. Then, the color values were measured in the CIELab color space using a dental spectrophotometer on a white background, with all samples centered, where

- L represents lightness (i.e., a grayscale from black to white)
- a represents chromaticity from green (negative) to red (positive)
- b represents chromaticity from blue (negative) to yellow (positive)

One advantage of this system is that it displays color differences as a single unit, which can be related to the visual perception system and clinical applications.^22,23^

 All the samples were evaluated according to the following formula:


$$\Delta E={\left[{\left(L2-L1\right)}^{2}+{\left(\alpha 2-\alpha 1\right)}^{2}+{\left(b2-b1\right)}^{2}\right]}^{1/2}$$

Where L1, a1, and b1 represent the values corresponding to the control group, and L2, a2, and b2 represent the values after applying HF acid etching and sandblasting surface preparations.^24^

###  Statistical Analysis

 SPSS 27 was used for data analysis. Data normality was evaluated using the Kolmogorov–Smirnov test; skewness and kurtosis were evaluated using the Shapiro–Wilk test. An independent t-test was used to compare two groups (when data were normally distributed), and one-way ANOVA was used to compare color differences based on the type of surface preparation between the study groups. If the parametric conditions were not met, the Mann-Whitney U test and the Kruskal-Wallis test were used, respectively. The significance level in all tests was set at 5%.

## Results

 In each test, if *P* > 0.05, the data can be considered to have a normal distribution. If skewness and kurtosis values fall within the range of ± 1, the data can be considered to have a normal or near-normal distribution. Overall, the statistical test results indicated that the data were normally distributed.

 Descriptive statistics related to color indices are presented in Tables 1 to 3 and Figures 4 to 7. The mean, standard deviation, minimum, and maximum color values across the three groups showed that the greatest color difference occurred in the sandblasting group, and L values were also lower in the sandblasting group than in the other two groups.

**Table 1:** Descriptive results of color variables in the study groups

**Group**	**Variable**	**Mean**	**Min**	**Max**	**SD**
Control	L	77.85	76.43	78.94	0.65
	a	2.31	2.06	2.63	0.13
	b	22.50	21.24	23.41	0.62
	ΔE	0.77	0.6	1.46	0.43
Etching	L	77.25	76.88	77.80	0.26
	a	2.30	2.20	2.40	0.05
	b	22.46	21.90	22.90	0.29
	ΔE	0.67	0.28	1.04	0.22
Sandblasting	L	76.12	75.70	76.50	0.23
	a	2.19	2.10	2.25	0.04
	b	21.91	21.50	22.30	0.22
	ΔE	184	1.44	2.33	0.27

**Table 2:** Results of one-way ANOVA statistical test to examine the relationship between the study groups

**Group 1**	**Group 2**	**Mean ΔE group 1**	**SD group 1**	**No. of samples group 1**	**Mean ΔE group 2**	**SD group 2**	**No. of samples group 2**	**Mean difference between the two groups**	**Mean SD**	***P*** **-value**	**95% confidence interval**	
											**Upper**	**Lower**
Control	Etching	0.772	0.434	22	0.672	0.220	22	+ 0.101	0.097	0.904	0.338	-0.137
Control	Sandblasting	0.772	0.434	22	1.836	0.266	22	-1.063	0.097	< 0.001	-0.826	-1.301
Etching	Sandblasting	0.672	0.220	22	1.836	0.266	22	-1.164	0.097	< 0.001	-0.926	-1.401

**Table 3:** Results of one-way ANOVA statistical test to examine the effect of zirconia surface preparation type on color components

**Variable**	**Group 1**	**Group 2**	**Mean Group 1**	**SD Group 1**	**Mean Group 2**	**SD Group 2**	**Mean difference between the two groups**	**Std Error**	***P*** **-value**	**95% confidence interval**	
										**Upper**	**Lower**
L	Control	Etching	77.85	0.65	77.25	0.26	+ 0.592	0.127	< 0.001	0.905	0.279
	Control	Sandblasting	77.85	0.65	76.12	0.23	+ 1.726	0.127	< 0.001	2.039	1.413
	Etching	Sandblasting	77.25	0.26	76.12	0.23	+ 1.135	0.127	< 0.001	1.448	0.822
a	Control	Etching	2.31	0.13	2.30	0.05	+ 0.011	0.026	1.000	0.074	-0.052
	Control	Sandblasting	2.31	0.13	2.19	0.04	+ 0.120	0.026	< 0.001	0.183	0.057
	Etching	Sandblasting	2.30	0.05	2.19	0.04	+ 0.109	0.026	< 0.001	0.172	0.046
b	Control	Etching	22.50	0.62	22.46	0.29	+ 0.035	0.125	1.000	0.341	-0.272
	Control	Sandblasting	22.50	0.62	21.91	0.22	+ 0.589	0.125	< 0.001	0.895	0.282
	Etching	Sandblasting	22.46	0.29	21.91	0.22	+ 0.554	0.125	< 0.001	0.860	0.248

**Figure 4:**
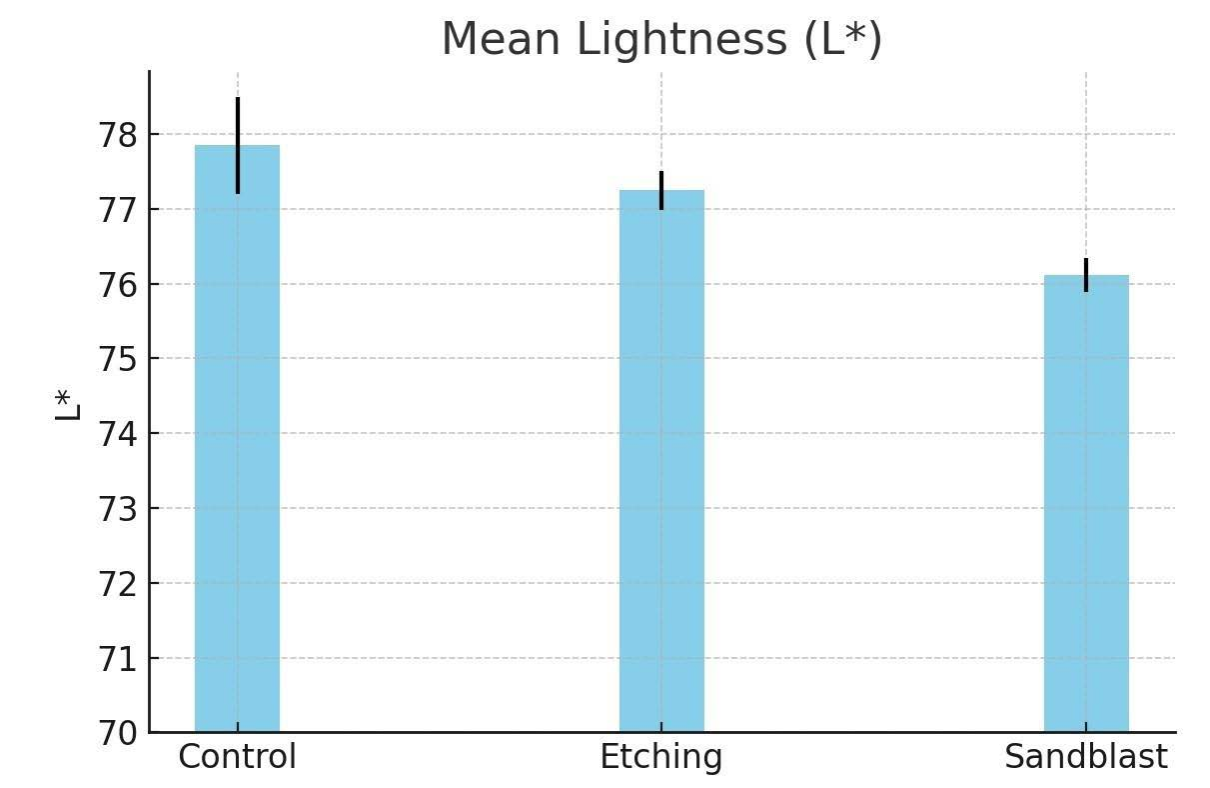
Bar chart comparing mean lightness (L) based on surface preparation method

**Figure 5:**
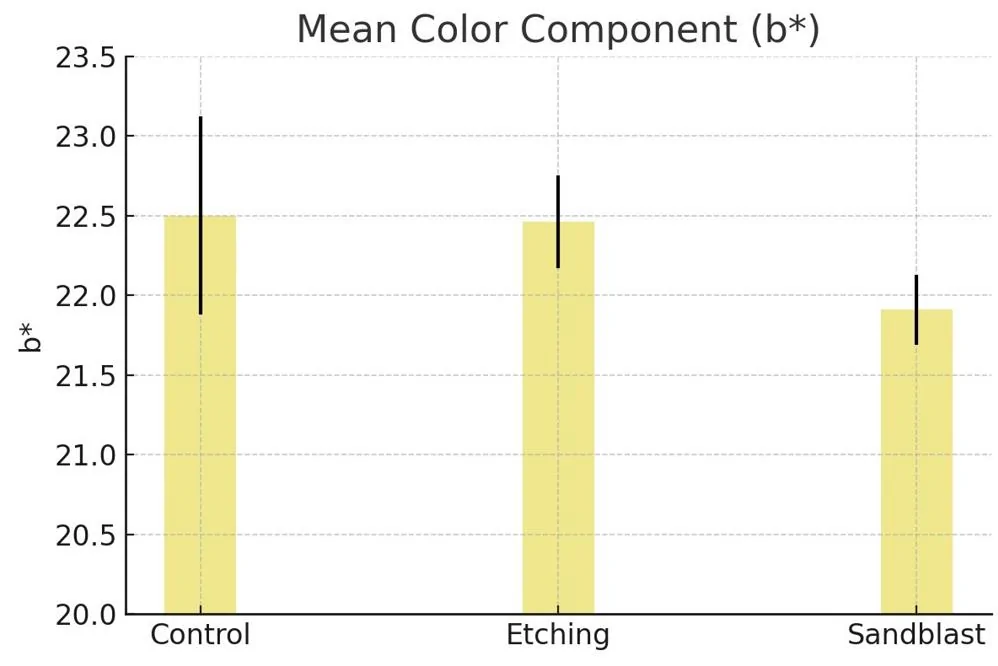
Bar chart comparing the mean color index (b) based on surface preparation method

**Figure 6:**
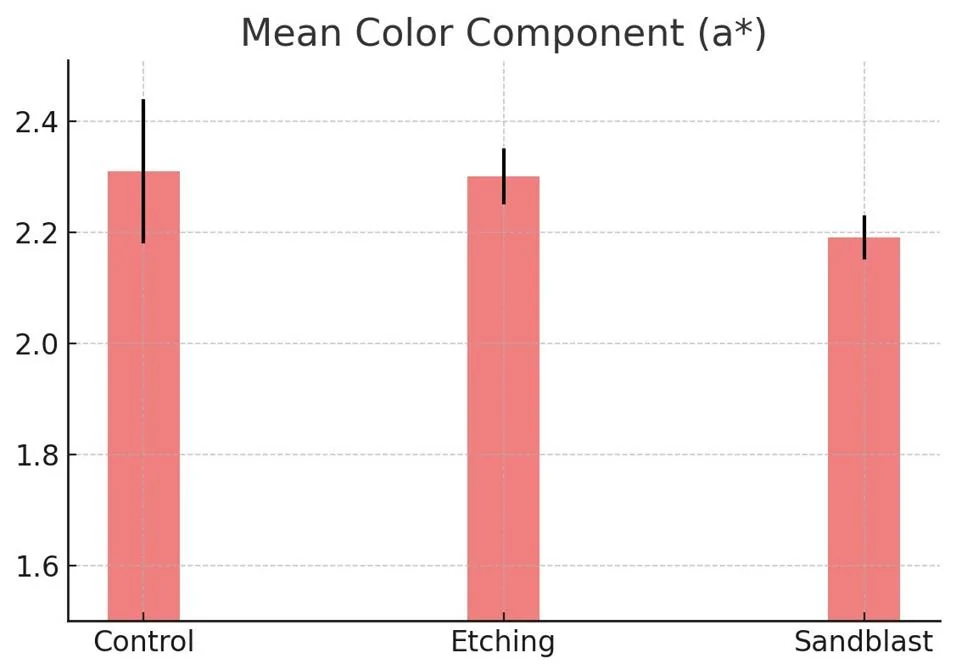
Bar chart comparing mean color index (a) based on surface preparation method

**Figure 7:**
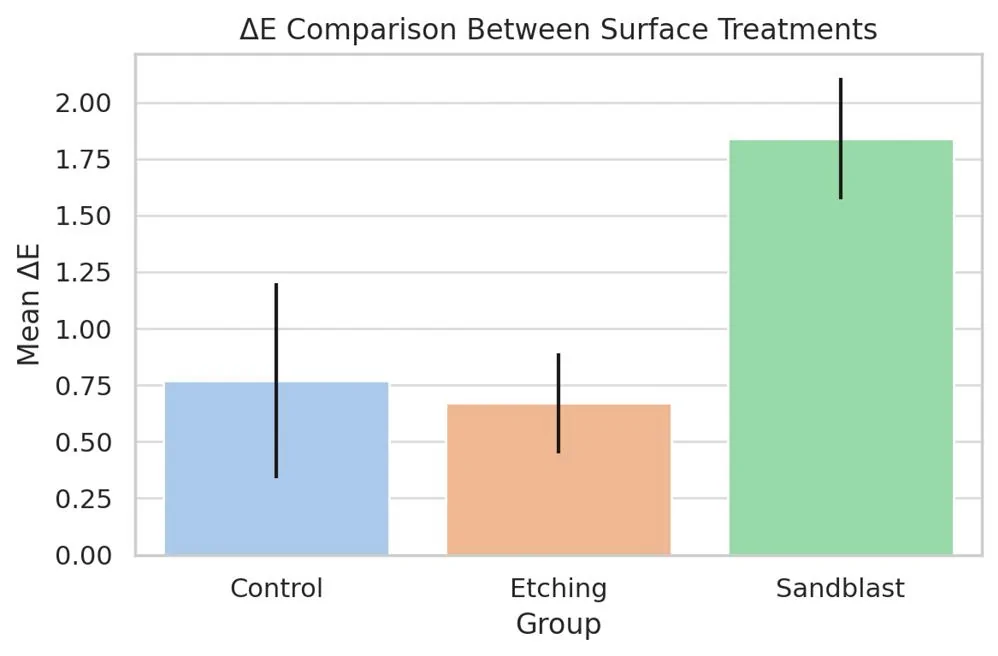
Mean ΔE based on the zirconia surface preparation method

 One-way ANOVA was used to compare the final color difference (ΔE) across the three study groups (Table 2). The results showed that the mean ΔE was significantly different across the three groups (F = 89.25, *P* < 0.001). To more precisely identify the differences between groups, a post hoc Bonferroni test was performed. The results showed that the mean difference in ΔE was not significantly different between the control and etching groups (*P* = 0.904); however, the differences between the control and sandblasting groups (*P* < 0.001) and between the etching and sandblasting groups (*P* < 0.001) were significant. These results indicated that sandblasting surface preparation significantly increased the color difference of restorations compared to the other two methods and decreased color uniformity. In contrast, the etching method did not produce a significant change compared to the control group.

 To further examine the effect of zirconia surface preparation type on color components (lightness L, red-green spectrum a, and yellow-blue spectrum b), one-way ANOVA was used for the three groups: control, etching, and sandblasting (Table 3). The results for all three variables showed significant differences between groups. To more precisely identify groups with differences, a post hoc Bonferroni test was applied. For the L component, the mean difference across all groups was statistically significant (control < etching < sandblasting, all *P* < 0.001). Regarding variables a and b, there was no significant difference between the control and etching groups (*P* = 1.000), but the sandblasting group showed significant differences from both other groups (*P* < 0.001). These results indicated that sandblasting-type surface preparation had the greatest effect in reducing L and changing the color components a and b. In contrast, HF acid etching preparation had no considerable effect compared to the control group, and color values in these two groups remained approximately similar.

## Discussion

 One of the main challenges in the clinical application of zirconia is its limited light transmission and the potential influence of its surface on the final color of the restoration. Minor changes in the core’s surface properties can affect the final color perception by the patient or the dentist. Therefore, surface preparation methods such as sandblasting and acid etching, which are used to improve the bond between the porcelain layer and zirconia, may affect how light is reflected, the degree of light transmission, and consequently the visual perception of the final color.

 According to the present study, the sandblasting group exhibited significant reductions in lightness (L) and other color parameters. One possible reason for this finding is the physical nature of sandblasting: the application of abrasive particles (usually alumina) to the surface roughens, increases porosity, and alters the microstructure of the zirconia surface.^25,26^ These changes not only improve mechanical bonding between the core and porcelain but also likely reduce light transmission and increase irregular light reflection by reducing surface uniformity and increasing light scattering.^27^

 In contrast, in the etching group, despite the use of a composite solution containing hydrofluoric acid and nitric acid, no significant change in ΔE was observed compared to the control group because, unlike ceramics such as lithium disilicate, acid etching on zirconia usually has a negligible effect on the surface,^28,29^ as zirconia lacks a glassy phase that acid can easily dissolve, causing surface roughening.^30^ Therefore, under the conditions used in this study, the surface changes resulting from etching were likely so limited that they did not significantly affect the light transmission pathway and the final color.

 The findings of this study on the effect of sandblasting surface preparation on changes in final restoration color are consistent with previous research. Kim et al.^27^ reported that surface preparation with alumina particles results in greater surface roughness and significant changes in the optical properties of zirconia, which can ultimately affect the final restoration color. Also, Atsu et al.^25^ reported that sandblasting increases light scattering and reduces its transmission from the core to the porcelain layer, resulting in decreased lightness and increased ΔE. Vichi et al.^31^ showed that methods that roughen the core surface directly affect the color match of the restoration and can lead to observable color differences in clinical conditions. Additionally, Kern and Wegner^29^ noted that rougher preparations, such as sandblasting, although they strengthen the porcelain bond, may adversely affect the final optical properties.

 On the other hand, the absence of a significant difference between the etching and control groups in the present study is also consistent with some previous reports. For example, Usumez et al.^32^ showed that acid etching has no significant effect on surface roughness or color changes of Y-TZP zirconia.

 The results of this research have significant implications for dentists and dental technicians in selecting appropriate methods for zirconia surface preparation and color matching of restorations. Especially in anterior regions, where esthetics and color matching play an important role in patient satisfaction, the choice of surface preparation method can significantly influence the final appearance of restorations.

## Suggestions

1. Long-term studies on the effects of surface preparation on the color and optical properties of zirconia, particularly concerning aging, fatigue, and corrosion, can provide further insight into the durability of the results of this research.
2. Research on different types of porcelain used for veneers, especially regarding color matching and their effects on optical properties, can help optimize material selection for different applications.
3. Research investigating new methods for zirconia surface preparation can help identify optimal techniques to improve optical properties and the porcelain bond to zirconia. Methods such as nanocoating and laser-based technologies can yield better results than traditional methods like sandblasting and etching.
4. Research comparing different types of zirconia with varying compositions and structures can help determine the best type for esthetic and functional applications.

## Conclusion

 This study demonstrated that sandblasting-based surface preparation increased color difference (ΔE) and decreased lightness (L). In contrast, the etching and control groups showed no significant difference in color characteristics. These findings indicate that surface preparation methods must be carefully selected, as changes in optical properties can directly affect the treatment outcome.

## Competing Interests

 The authors declare that they have no competing interests.

## Data Availability Statement

 The datasets used and/or analyzed during the current study are available from the corresponding author on reasonable request.

## Ethical Approval

 Ethical approval was granted by the Ethics Committee of Tabriz University of Medical Sciences (IR.TBZMED.VCR.REC.1403.053), and all experimental procedures were conducted in accordance with the approved protocol.
